# OSW‐1 inhibits tumor growth and metastasis by NFATc2 on triple‐negative breast cancer

**DOI:** 10.1002/cam4.3196

**Published:** 2020-06-08

**Authors:** Xiaorong Ding, Yumei Li, Jun Li, Yongmei Yin

**Affiliations:** ^1^ Department of Oncology The First Affiliated Hospital of Nanjing Medical University Nanjing China; ^2^ Department of Oncology The Affiliated Huaian No.1 People’s Hospital of Nanjing Medical University Huaian China

**Keywords:** apoptosis, epithelial‐mesenchymal transition, metastasis, NFATc2, OSW‐1, triple‐negative breast cancer

## Abstract

OSW‐1 is a natural compound extracted from the bulbs of *Ornithogalum saundersiae* in 1992. It has been shown strong antitumor activities in various cancer cells. However, the effects of OSW‐1 on tumor growth and metastasis in breast cancer are still poorly understood. In our research, we showed that OSW‐1 had a strong anticancer effect on breast cancer cells, but lower toxicity to normal cells. Accordingly, it also revealed significant inhibition of tumor growth by OSW‐1 in xenograft model. In addition, we performed Annexin V/PI‐labeled flow cytometric assay and TUNEL assay and showed that OSW‐1 inhibited tumor growth by inducing apoptosis. Furthermore, we carried out transwell assays and found that OSW‐1 significantly repressed the migratory and invasive capabilities of triple‐negative breast cancer (TNBC) cells via mediating epithelial‐mesenchymal transition. Besides, OSW‐1 also could inhibit metastasis in an orthotopic model and resulted in a longer survival compared with control group. Finally, we performed RNA‐sequencing and cellular functions to investigate the molecular mechanism of how OSW‐1 inhibits TNBC, and identified NFATc2 may as a pivotal factor for OSW‐1‐mediated effects on cell death, tumor growth, invasion, and migration.

## Graphical abstract

OSW‐1, as a natural compound, could inhibit tumor growth by activating apoptosis and supress metastasis via epithelial‐mesenchymal transition in triple‐negative breast cancer (TNBC) both in vitro and in vivo. Besides, to investigate the molecular mechanism of how OSW‐1 inhibits TNBC, we performed RNA sequencing and cellular functions and considered that NFATc2 may act as a pivotal role in OSW‐1‐induced cell death, tumor growth, invasion, migration.

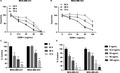

## 1 INTRODUCTION

Globally, breast cancer has the highest morbidity and mortality among all types of cancer in women.^1^ Triple‐negative breast cancer (TNBC), one particular phonetype in breast cancer, has the poorest prognosis with a medium overall survival (OS) rarely reaching 12 to 18 months.^2^, ^3^, ^4^, ^5^, ^6^ Chemotherapy remains the main treatment in TNBC, many well‐known chemotherapeutic drugs, such as adriamycin, docetaxel, and cisplatin, are used to treat breast cancer.^7^ Although all kinds of advancements in chemotherapy for curing breast cancer have been made, the efficacy of TNBC chemotherapy is limited because of the lack of specific therapeutic molecular targets in TNBC.^8^ Thus, the discovery of developing optimal therapeutic strategies with lower toxicity is a challenge for treating TNBC.

OSW‐1(3β,16β,17α‐trihydroxycholest‐5‐en‐22‐one‐16‐O‐(2‐O‐4‐methoxybenzoyl‐β‐D‐xylopyranosyl)‐(1 → 3)‐(2‐O‐acetyl‐α‐L‐arabinopyranoside) is a steroidal glycoside isolated from the bulbs of *Ornithogalum saundersiae* by Kubo et al in 1992.^9^ Recently, several findings have revealed that OSW‐1 could kill various cancer cells, such as colon cancer cells, hepatocellular carcinoma, leukemia, and so on.^10^, ^11^, ^12^ Furthermore, it was found, in the 60‐cell in vitro screening by the National Cancer Institute, OSW‐1 not only shows a considerable anticancer activity with an average IC50 of 0.78 nmol/L but also displays a 10‐100 times selective cytotoxicity against normal cells.^13^ But there has been no report of exhaustive mechanism about such selectivity.^14^ Mechanistically, OSW‐1 may induce calcium‐dependent apoptosis by damaging the mitochondrial transmembrane and cellular homeostasis.^15^ The synthesis of OSW‐1 was done in 1999, however, its antitumor effect is extremely complicated and remains largely unclear.

To interrogate the cytotoxicity of OSW‐1 in breast cancer and its anticancer mechanism, we investigate how OSW‐1 influences the tumor growth and metastasis in breast cancer, especially in TNBC. In our research, we performed transwell assays to quest the effect of OSW‐1 on TNBC cell migration and invasion.In addition, we implanted orthotopic breast tumors in the mice to test the effect of OSW‐1 on tumorigenesis. Furthermore, we explored how OSW‐1 affects proliferation and metastasis via measuring the associated markers in breast cancer cells and tissues. Finally, to study the molecular mechanism of how OSW‐1 inhibits TNBC, we performed RNA‐sequencing and cellular functions and considered that NFATc2 may mediate the effect of OSW‐1 on cell death, tumor growth, invasion, and migration.

## 2 MATERIALS AND METHODS

### 2.1 Cell culture

The human breast cancer cell lines (MCF‐7, BT474, T47D, ZR‐75‐1, SKBR3, MDA‐MB‐231, and MDA‐MB‐453) and normal endothelial cell line (MCF/10A) were purchased from Cell Bank of Shanghai Institute of Chinese Academy of Sciences. Mouse breast cancer cell line 4T1 was supplied by Nanjing Kebai Biotechnology Co., Ltd. SKBR3 and 4T1 cells were incubated in RPMI‐1640 (Gibco) with 10% FBS (Gibco). MCF/10A cells were maintained with Endothelial Cell medium (Sciencell). The other cells were maintained in DMEM (Gibco) with 10% FBS (Gibco). OSW‐1 was supplied by Changbai Mountain Institute of Traditional Chinese Medicine.^12^

### 2.2 Cytotoxicity assay

Human breast cancer and normal endothelial cells (1 × 10^4^ cells/well) were inoculated into 96‐well plates and incubated overnight. Cells were treated with DMSO or various concentrations of OSW‐1 for 72 hours, then, the cytotoxicity was detected with cell count kit 8 (CCK8) (Dojindo). IC50 was defined as the concentration of OSW‐1 to reduce the viable cells by 50% relative to control cells.

### 2.3 Cell proliferation

Cell proliferation analysis was measured by CCK8 (Dojindo). To confirm cell viability treated with OSW‐1, TNBC cells (MDA‐MB‐231 and MDA‐MB‐453, 1 × 10^4^ cells/well) were incubated in 96‐well plates overnight and then treated with different concentrations of OSW‐1 for 24, 48, and 72 hours at 37°C in 5% CO_2_.

### 2.4 Flow cytometry assay

MDA‐MB‐231 and MDA‐MB‐453 cells (2 × 10^5^ cells/well) were inoculated in 6‐well plates and treated with OSW‐1 (100 ng/mL) for 24 hours. The apoptotic ratio of cells was determined by staining with Annexin V‐FITC/PI (Invitrogen; Thermo Fisher Scientific, Inc). Cells subsequently were measured by flow cytometer (BD Biosciences Inc).

### 2.5 TUNEL assay

A TUNEL kit (Roche) was used to evaluate apoptosis. Specifically, TNBC cells (1 × 10^4^ cells/well) were cultured on the confocal dishes overnight and treated with 50 ng/mL OSW‐1 for 24 hours. The cells were fixed with 4% paraformaldehyde for 30 minutes, then incubated with PBS containing 0.2% Triton X‐100 for 10 minutes. After washed with PBS, the cells were added with TUNEL reaction solution following the manufacturer's protocols. The number of TUNEL positive cells were counted using an inverted fluorescence microscope and cells were scored in five randomly chosen fields under a magnification of 200× per sample.

### 2.6 Transwell assay

TNBC cells (1 × 10^4^ cells/well) with or without OSW‐1 (6.25 ng/mL) were seeded in transwell chambers (Corning) with or without matrigel mix (BD Biosciences). After overnight incubation, nonmigrated or noninvaded cells were removed from the upper part of the chambers. Then, cells were stained with 1% crystal violet and counted using a microscope.

### 2.7 Western blotting analysis

Western blotting was carried out using cell lysates. After separated on 15% SDS‐PAGE gels, cell extracts were transferred into polyvinylidene fluoride membranes. Antibodies for proteins used in this analysis were anti‐caspase‐3, anti‐cleaved caspase‐3, anti‐Bcl‐2, anti‐PARP, anti‐cleaved PARP, and anti‐Vimentin (Cell Signaling Technology), anti‐E‐Cadherin (BD Biosciences), anti‐NFATc2 (Santa Cruz Biotechnology), and anti‐β‐actin (Sigma). Rabbit or mouse (HRP)‐linked secondary antibodies were supplied by Cell Signaling Technology.

### 2.8 Quantitative real‐time PCR

Total RNA from cells was isolated by Trizol reagent (Invitrogen). After identifying its purity and integrity, total of 1 μg RNA was transcribed into complementary DNA using Reverse Transcription Supermix (Takara). Quantitative real‐time PCR (RT‐PCR) was performed using SYBR Green PCR master mix (Takara). The sequences of the primers used for amplification were as follows: MATR3 forward: 5′‐TCTTGGGGGACCAGCAGTTGGA‐3′, MATR3 reverse: 5′‐GCTAGTTTCCACTCTGCCTTTCTGC‐3′; NFATc2 forward: 5′‐GAGGGGCTGTCAAAGCTCC‐3′, NFATc2 reverse: 5′‐ACAGTTTTCCCCGTGATTCGG‐3′; FBXW11 forward: 5′‐GTGGGATGTGAACACGGGTGA‐3′, FBXW11 reverse: 5′‐CGTAAAGTGATGTCGGTCGCAG‐3′; ZBED6 forward: 5′‐CAAGACATCTGCAGTTTGGAATTT‐3′, ZBED6 reverse: 5′‐TGTCGTTGAAGTGTTGAAGTTCCTA‐3′; FAT1 forward: 5′‐GACGCAGACATCCGCTCTAA‐3′, FAT1 reverse: 5′‐AAACAGCTTGCTCCTCACGA‐3′; PTBP3 forward: 5′‐ACAGCTAATGGGAATGACAGCA‐3′, PTBP3 reverse: 5′‐CTGGCTTCGAAGGTGAGGAG‐3′; MYC forward: 5′‐AAAACCAGCAGCCTCCCGCGA‐3′, MYC reverse: 5′‐AATACGGCTGCACCGAGTCGT‐3′; ZBTB38 forward: 5′‐GGTGTGACATCTCATGTGCATT‐3′, ZBTB38 reverse: 5′‐AAGGCCCCACCGACATCTTA‐3′; ASXL2 forward: 5′‐GAATCCAGGTGCGAAAAGTAC‐3′; ASXL2 reverse: 5′‐GATGGAGACTGGAAAACGAGC‐3′; HRH1 forward: 5′‐AGATGTGTGAGGGGAACAGG‐3′, HRH1 reverse: 5′‐TACAGCACCAGCAGGTTGAG‐3′; GAPDH forward: 5′‐TGACTTCAACAGCGACACCCA‐3′, GAPDH reverse: 5′‐ACCCTGTTGCTGTAGCCAAA‐3′.

### 2.9 shRNA packaging and establishment of stable cell lines

The scrambled shRNA and human NFATc2 shRNA (shNFATc2) were obtained from Santa Cruz Biotechnology and transfected into 293T cells using Lipofectamin 2000 (Invitrogen) following the manufacturer's instructions. Six hours after transfection, the medium was changed with refresh complete medium and incubated for 48 hours. Supernatant was collected and filtered with 0.45 µm membrane filter. Viruses were stocked at −80°C until transfection in TNBC cells.

### 2.10 Immunofluorescence analysis

TNBC cells (1 × 10^4^) were seeded on the confocal dishes overnight and treated with OSW‐1 (6.25 ng/mL) for 12 hours. Next, cells were washed three times with PBS and fixed with 4% formaldehyde for 30 minutes on ice. After that, cells were punched with 0.2% Triton X‐100 for 10 minutes at room temperature. Then, cells were blocked using 5% BSA for 1 hour at room temperature and incubated with primary antibodies (anti‐E‐Cadherin, anti‐Vimentin; Cell Signaling Technology) at 4°C overnight. Next day, cells were washed three times in PBS and then incubated with secondary antibodies for 2 hours at room temperature (Goat anti‐Rabbit, Alexa Fluor 488, ab150077; Fluor 594, ab150080) for 2 hours at room temperature. Cells were washed three times with PBS and added with DAPI (Beyotime Biotechnology, China) for 5 minutes. Finally, an immunofluorescence microscopy (Olympus) was used to observe the cells.

### 2.11 Animal models

The animal experiments are approved by Animal Care Committee of Nanjing Medical University (Approval no. IACUC‐1903022). For xenograft model, 10 four‐week‐old female SCID mice were purchased from Beijing Vital River Laboratory Animal Technology, Co., Ltd. MDA‐MB‐231 cells (5 × 10^6^) were suspended in 50 μL serum‐free DMEM medium and subcutaneously injected into mammary fad pat (MFP) of SCID mice. When the tumor became palpable, the SCID mice were randomized to two groups of five mice each: Control group, PBS (100 μL, daily); OSW‐1‐treated group, OSW‐1 (0.01 mg/kg diluted in 100 μL PBS, daily) for 20 days. Mice weights and tumor sizes were recorded every 4 days. Formula of tumor volume was as follows: [(length) × (width) × (length + width/2) × 0.526 = volume]. After 20 days treatment, the subcutaneous tumors were excised and weighed.

For orthotopic model, 40 six‐week‐old female BALB/c mice were divided into two groups: Control (PBS, 100 μL) and OSW‐1 treatment group (OSW‐1, 0.01 mg/kg diluted in 100 μL PBS). 4T1 cells (5 × 10^4^) were suspended in 50 μL DMEM medium and subcutaneously injected into the MFP. We removed the tumors when the largest tumor in control group reached 1.0 cm, continued injecting OSW‐1 for 1 week. After surgery resection for 10 days, 10 mice in control group and 10 mice in OSW‐1‐treated group were sacrificed, collected lung tissues from mice and counted the number of cancer metastases on lung tissue. Another 20 mice (10 mice in control group and 10 mice in OSW‐1‐treated group) were used for survival analysis.

For knockdown NFATc2 model, stable controlled MDA‐MB‐231 cells (5 × 10^6^ cells) were injected into MFP of five SCID mice and stable NFATc2‐depleted MDA‐MB‐231 cells (5 × 10^6^ cells) were injected into MFP of 10 SCID mice. When the tumors turned palpable, the mice were randomized into three groups: (a) scr‐shRNA‐control group, intraperitoneal injection of PBS (100 µL, daily); (b) shNFATc2‐control group, intraperitoneal injection of PBS (100 µL, daily); (c) shNFATc2‐OSW‐1 group, intraperitoneal injection of (0.01 mg/kg diluted in 100 μL PBS, daily). After 20 days of treatment, tumors were excised and weighed.

### 2.12 Hematoxylin and eosin and immunohistochemistry

The tissues were fixed with 4% formaldehyde for 24 hours, embedded in paraffin and then underwent hematoxylin and eosin and immunohistochemistry. For immunohistochemical staining, slides were boiled in sodium citrate buffer (pH 6.0) for 20 minutes and incubated with 0.3% hydrogen peroxide (0.3% H_2_O_2_) for 20 minutes and then blocked with blocking solution (5% BSA in PBS) for 1 hour. Slides were incubated with primary antibodies (anti‐Ki67, abcam; anti‐PCNA, DaK; anti‐Vimentin, Cell Signaling Technology; anti‐E‐Cadherin, BD Biosciences) overnight at 4°C, followed by biotinylated secondary antibodies (anti‐Rabbit, DaKo; anti‐mouse, DaKo) and DAB detection.

### 2.13 Statistical analysis

The data in tumor volume were represented with mean ± SEM, the others were used mean ± SD. GraphPad Prism 7.0 was used to make all graphs. An independent *t* test was conducted to test statistical significance, and *P* < .05 was judged to indicate statistical significance.

## 3 RESULTS

### 3.1 OSW‐1 has a strong anticancer effect on different breast cancer cells, but not on normal cells

Previous studies have showed the cytotoxicity of OSW‐1 in pancreatic cancer, hepatocellular carcinoma, and colon cancer cells.^10^, ^11^, ^12^ We started our investigation using eight breast cancer lines from different subgroups and a normal endothelial cell line and determined the antitumor effects of OSW‐1 on these cells. As presented in Table 1, OSW‐1 could inhibit different subtypes of breast cancer cells with an IC50 nanomolar concentration, but had a lower toxicity to normal endothelial cells. These findings suggest that OSW‐1 shows an extremely strong cytotoxic effects on breast cancer cells, but lower toxicity to normal cells.

**Table 1:** IC50 values of OSW‐1 in various kinds of breast cancer cells and normal endothelial cells

Cells	IC50 (nmol/L)
Breast cancer cells	
MCF‐7	3.72 ± 0.78
T47D	5.92 ± 1.21
ZR‐75‐1	10.34 ± 0.07
BT474	6.54 ± 1.14
SKBR3	6.67 ± 0.13
MDA‐MB‐231	5.82 ± 2.35
MDA‐MB‐453	8.66 ± 0.19
HCC‐1937	11.12 ± 4.42
Normal endothelial cells	
MCF/10A	52.3 ± 8.72

Note

Data are present as the Mean ± SD. All the experiments were performed in triplicate.

**Figure 1:**
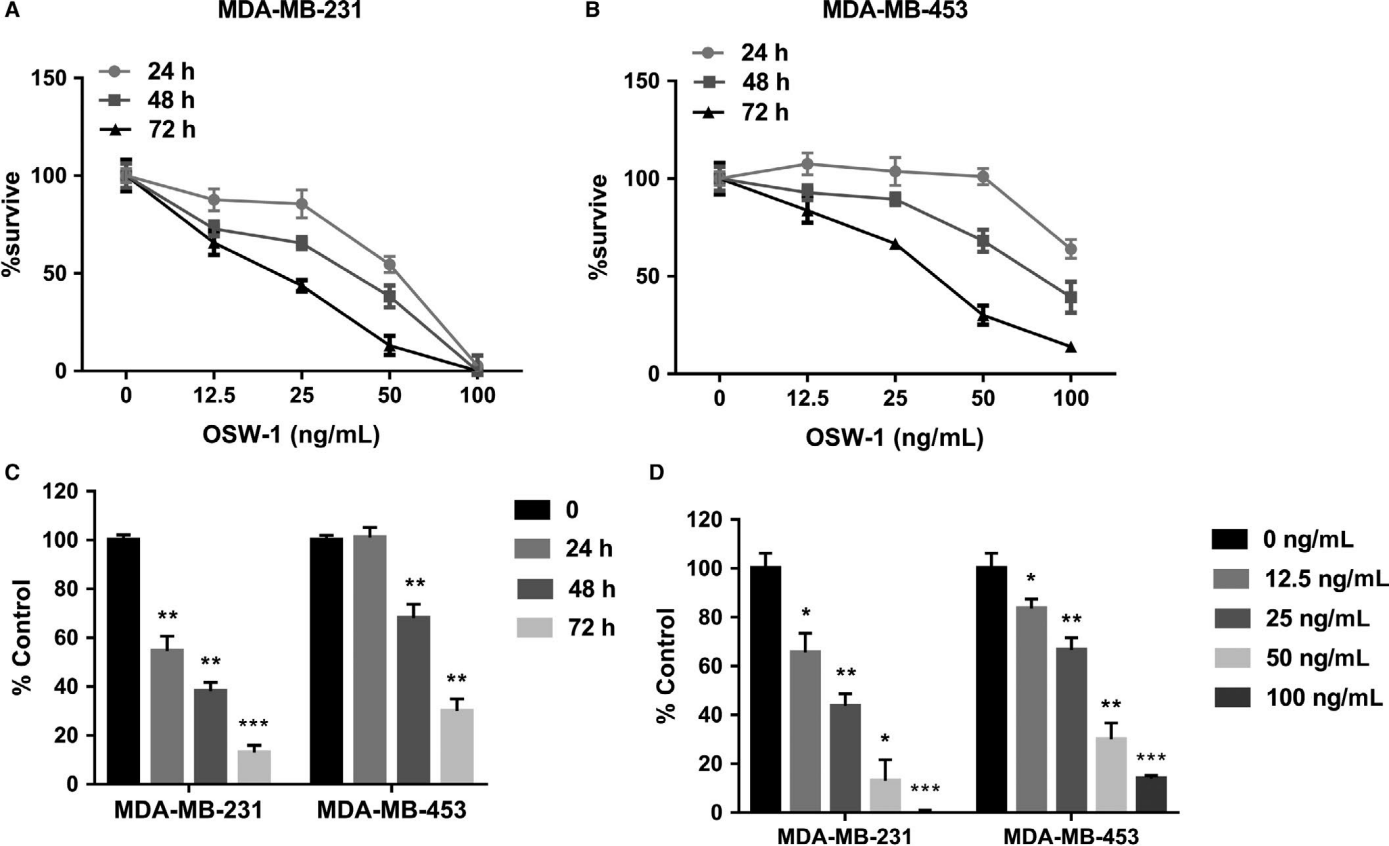
OSW‐1 inhibits cell proliferation in triple‐negative breast cancer (TNBC) cells. A,B, Cell count kit 8 (CCK8) assay showed that two breast cancer cells were sensitive to OSW‐1. TNBC cells were treated with OSW‐1 (12.5 to 100 ng/mL) for 24, 48, and 72 h. The CCK8 test was used to determine the proliferation of TNBC cells. C, OSW‐1 reduced TNBC cell proliferation in a time‐dependent manner. The cell viability was detected by CCK8 analysis after treatment with 50 ng/mL OSW‐1 for 24, 48, and 72 h. D, OSW‐1 reduced TNBC cell viability in a dose‐dependent manner. Cells were treated with OSW‐1 (12.5 to 100 ng/mL) for 72 h and detected by CCK8 analysis. *P < .05, **P < .01, ***P < .001, compared with control

TNBC is more malignant but has fewer therapeutic drugs, so we selected TNBC cell lines (MDA‐MB‐231 cells and MDA‐MB‐453 cells) for further study. OSW‐1 doses varying from 12.5 to 100 ng/mL significantly reduced TNBC cell viability in a dose‐ and time‐dependent manner (Figure 1A‐D).

### 3.2 OSW‐1 mediates apoptosis in TNBC cells in vitro

**Figure 2:**
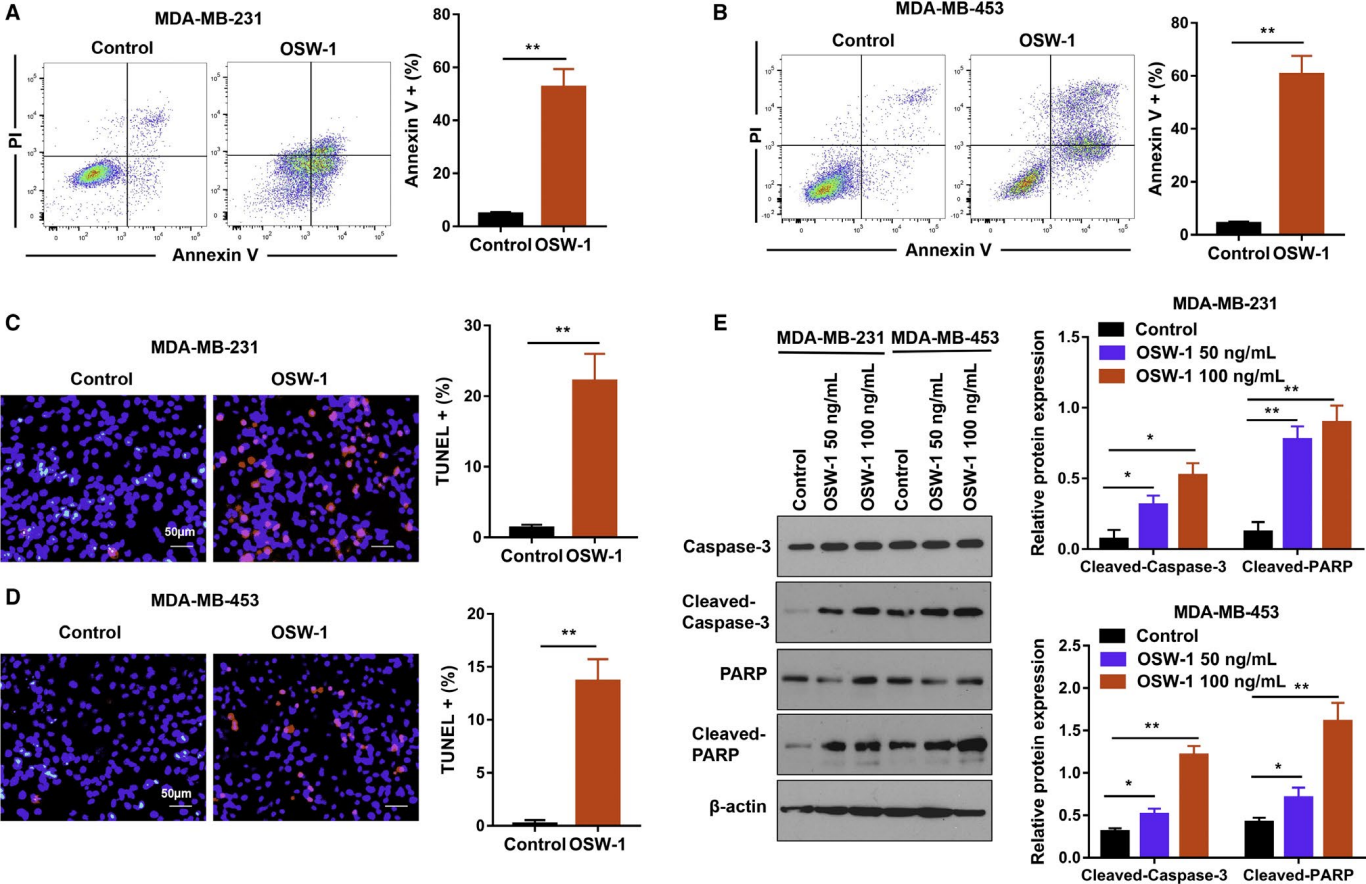
OSW‐1 mediates cell death in triple‐negative breast cancer cells. A,B, MDA‐MB‐231 and MDA‐MB‐453 cells were incubated with 100 ng/mL OSW‐1 for 24 h and then cell apoptosis was examined by flow cytometry assay. The results showed that apoptosis rate significantly increased in OSW‐1 groups compared with the nontreated groups. C,D, TUNEL staining also proved that the apoptosis rate of the OSW‐1 group (50 ng/mL) increased significantly than control group. Scale bars = 50 µm. E, MDA‐MB‐231 and MDA‐MB‐453 cells were treated with 50 ng/mL or 100 ng/mL OSW‐1 for 24 h, and apoptosis‐related proteins were measured by western blotting assay. The findings showed that cleaved PARP expression and cleaved caspase‐3 expression in protein levels increased in the OSW‐1 treatment groups (50 ng/mL, 100 ng/mL). Furthermore, the expression of cleaved PARP and cleaved caspase 3 was higher in 100 ng/mL OSW‐1‐treated cells than in 50 ng/mL OSW‐1‐treated cells. *P < .05, **P < .01

After incubation with 100 ng/mL OSW‐1 for 24 hours, TNBC cell apoptosis was assessed using flow cytometry and our data showed that OSW‐1 induced more apoptotic cells (MDA‐MB‐231 vs MDA‐MB‐453 cells, 52.5% vs 60.5%) than control groups. Apoptosis ratio in OSW‐1 treatment groups was significantly elevated compared with control groups (Figure 2A,B). Moreover, we performed TUNEL assay to further confirm whether OSW‐1 mediates cell death through apoptosis. Similarly, our data suggested that OSW‐1 groups showed more apoptotic cells than control groups both in MDA‐MB‐231 and MDA‐MB‐453 cells (Figure 2C,D). Collectively, these data demonstrated that OSW‐1 induced apoptosis in TNBC cells. Finally, TNBC cells treated with OSW‐1 (50 ng/mL or 100 ng/mL) for 24 hours were applied to measure the expression of apoptosis‐associated proteins by western blotting analysis. Compared with non‐OSW‐1‐treated controls, both cleaved PARP and cleaved caspase 3 expressions in protein levels were increased in OSW‐1‐treated groups (Figure 2E). Furthermore, the overexpressed cleaved PARP and cleaved caspase 3 were found in 100 ng/mL OSW‐1‐treated cells than those in 50 ng/mL OSW‐1‐treated cells (Figure 2E). These dose‐dependent results demonstrated that apoptosis mediated by OSW‐1 was partly through activating caspase.

Altogether, these results indicate that OSW‐1‐mediated anticancer activity might be via apoptosis pathway.

### 3.3 OSW‐1 inhibits tumor growth of TNBC in vivo

**Figure 3:**
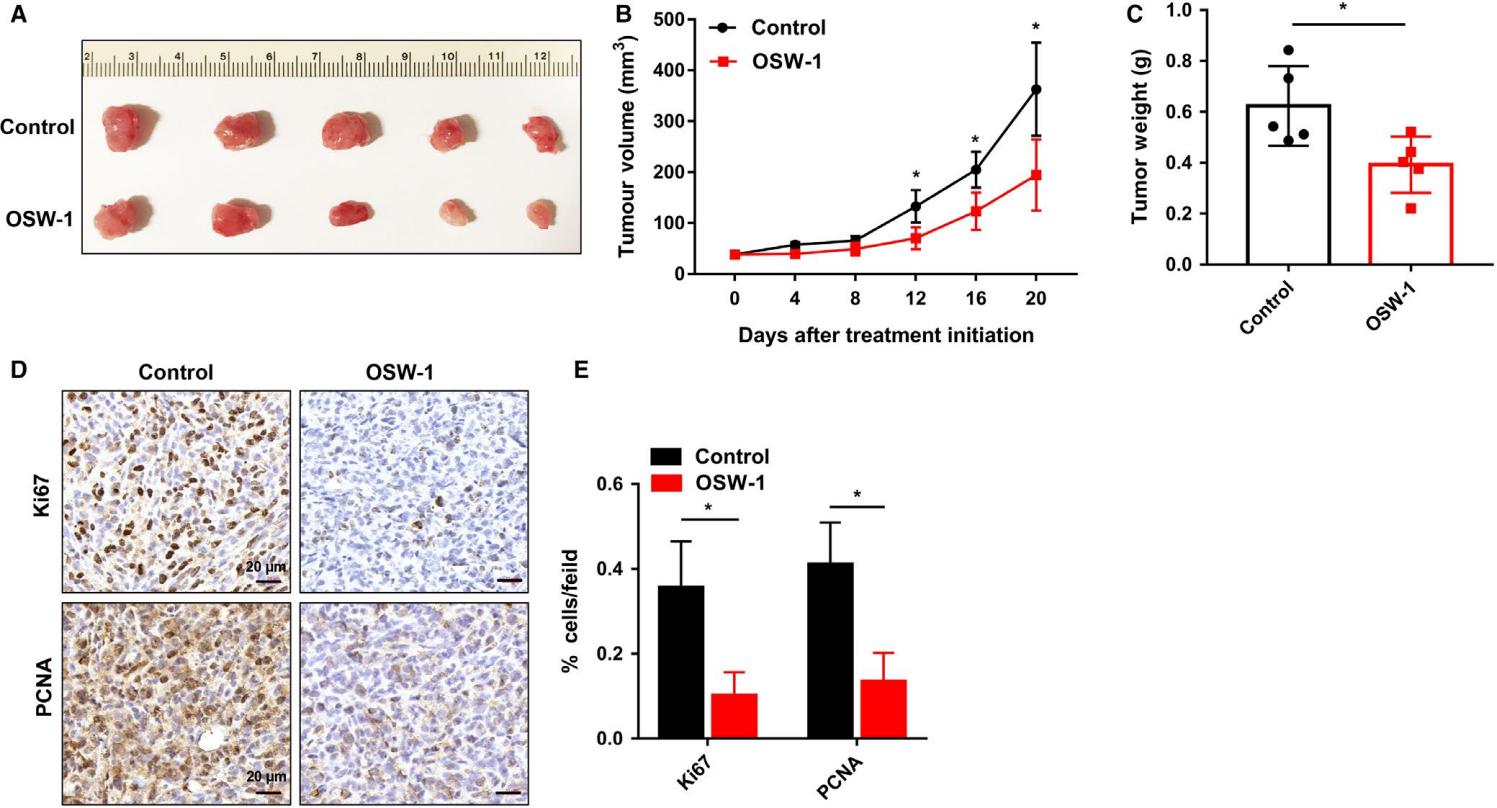
OSW‐1 suppresses tumor growth in vivo. A, Removing subcutaneous tumors after OSW‐1 treatment for 20 d. OSW‐1‐treated group showed smaller tumor size than nontreated group. B, Tumor volume was measured every 4 d. There was a great difference between OSW‐1 group and control group after the 12 d of OSW‐1 treatment, and then it became more and more apparent. C, Tumor weight analysis also showed lighter tumor weight in OSW‐1‐treated group than in nontreated group. D, The reduction of proliferation markers Ki‐67 and PCNA was observed in the treated group by immunohistochemistry assay. Scale bar = 20 µm in (D), *P < .05

Next, to confirm whether OSW‐1 could also repress TNBC in animal model, 5 × 10^6^ MDA‐MB‐231 cells were injected into the MFP of 4‐ to 6‐week‐old female SCID mice. We observed that the tumor sizes in OSW‐1 group were much smaller than those in control group from the 12th day (Figure 3A,B). Meanwhile, as shown in Figure 3C, tumor weights showed marked repression after treatment with OSW‐1 for 20 days (Figure 3C). Furthermore, tumor tissues were stained by immunohistochemistry assay with Ki67 and PCNA (Proliferating Cell Nuclear Antigen), which are markers of cell proliferation, and showed lower expression in OSW‐1‐treated xenografts compared with control group (Figure 3D,E). These findings indicate that OSW‐1 significantly suppresses tumor growth of TNBC in vivo.

### 3.4 OSW‐1 suppresses migration and invasion mediated by EMT in vitro

**Figure 4:**
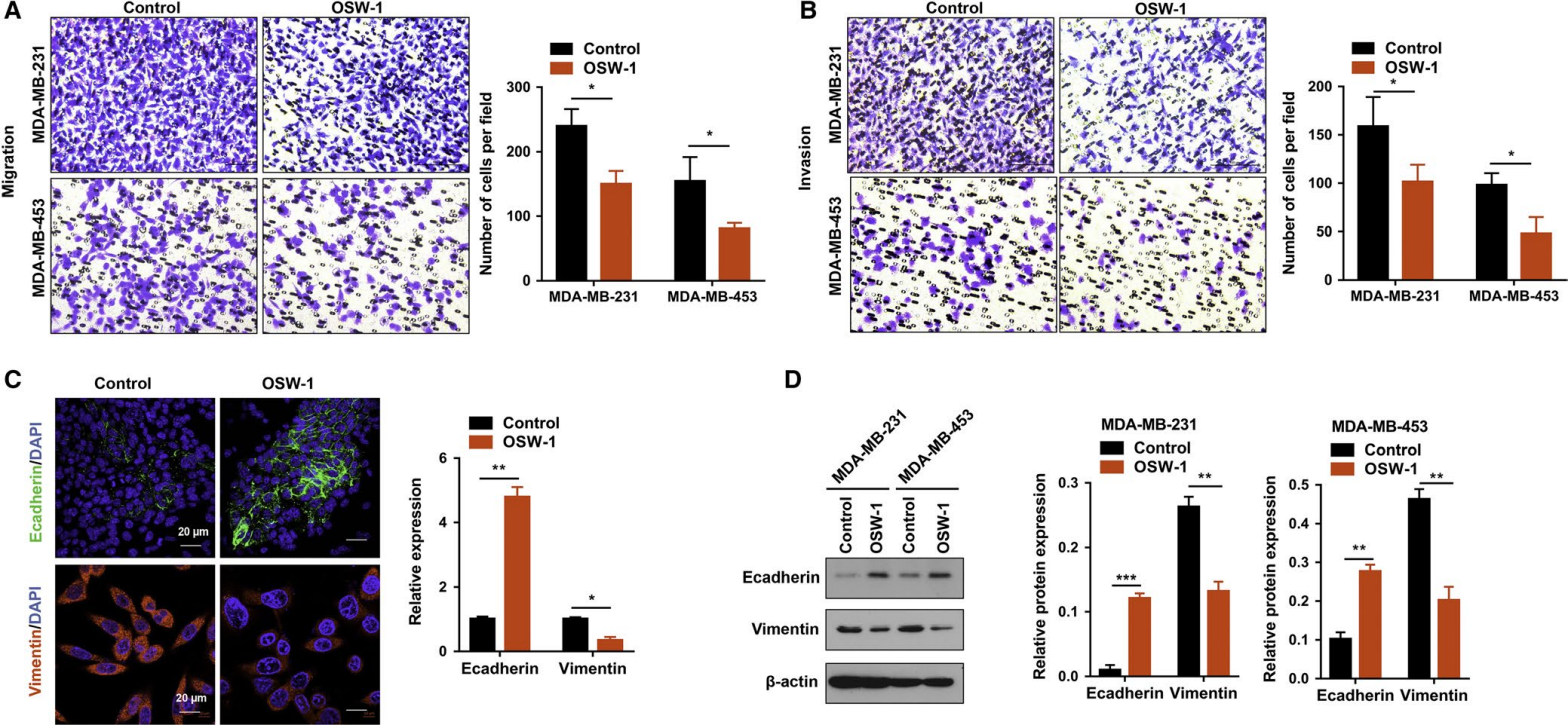
OSW‐1 reduces oncogenesis of breast cancer cells by affecting epithelial‐mesenchymal transition (EMT) transformation. A,B, MDA‐MB‐231 and MDA‐MB‐453 cells were added with 6.25 ng/mL OSW‐1 for 12 h. The number of cells that penetrating the transwell membrane was significantly reduced (*P* < .05), indicating that the ability of triple‐negative breast cancer cell migration and invasion was decreased after OWS‐1 treatment. C, Immunofluorescence assay showed that the expression of EMT‐related protein E‐cadherin increased while Vimentin decreased in the OSW‐1 treatment group. Scale bar = 20 µm. D, OSW‐1‐treated group showed significant elevation of E‐cadherin and reduction of Vimentin in protein levels by western blotting assay. *P < .05, **P < .01, ***P < .001

TNBC is particularly prone to distant metastasis, which results in a poor prognosis.^2^, ^3^, ^4^, ^5^, ^6^ So, we further examined how OSW‐1 affects the migration and invasion. Figure 1 showed that OSW‐1 at the concentration of 12.5 ng/mL and treated for 24 hours did not have the antitumor effect. We used lower concentration of OSW‐1 (6.25 ng/mL) to observe the effects on migration and invasion by OSW‐1. After treatment with OSW‐1 (6.25 ng/mL for 12 hours), the number of migrated TNBC cells was obviously decreased (Figure 4A). For invasion assay, OSW‐1‐treated groups exhibited inhibited invasive ability when compared with non‐OSW‐1 groups (Figure 4B). These findings suggest that OSW‐1 inhibits the migration and invasion of TNBC cells in vitro.

Recently, epithelial‐mesenchymal transition (EMT) has been known as a key mechanism for promoting the initiation of metastasis and supporting cancer cells with migratory and invasive properties.^16^, ^17^, ^18^ To test whether the repression of migration and invasion by OSW‐1 is depending on EMT, we detected EMT markers. As revealed by immunofluorescence and western blotting assays, when compared with control group, treatment with OSW‐1 showed elevation of E‐cadherin expression and reduction of Vimentin expression in protein levels (Figure 4C,D). Thus, these results suggest that OSW‐1 inhibits migration and invasion mediated by EMT in vitro.

### 3.5 OSW‐1 inhibits metastasis mediated by EMT in vivo

**Figure 5:**
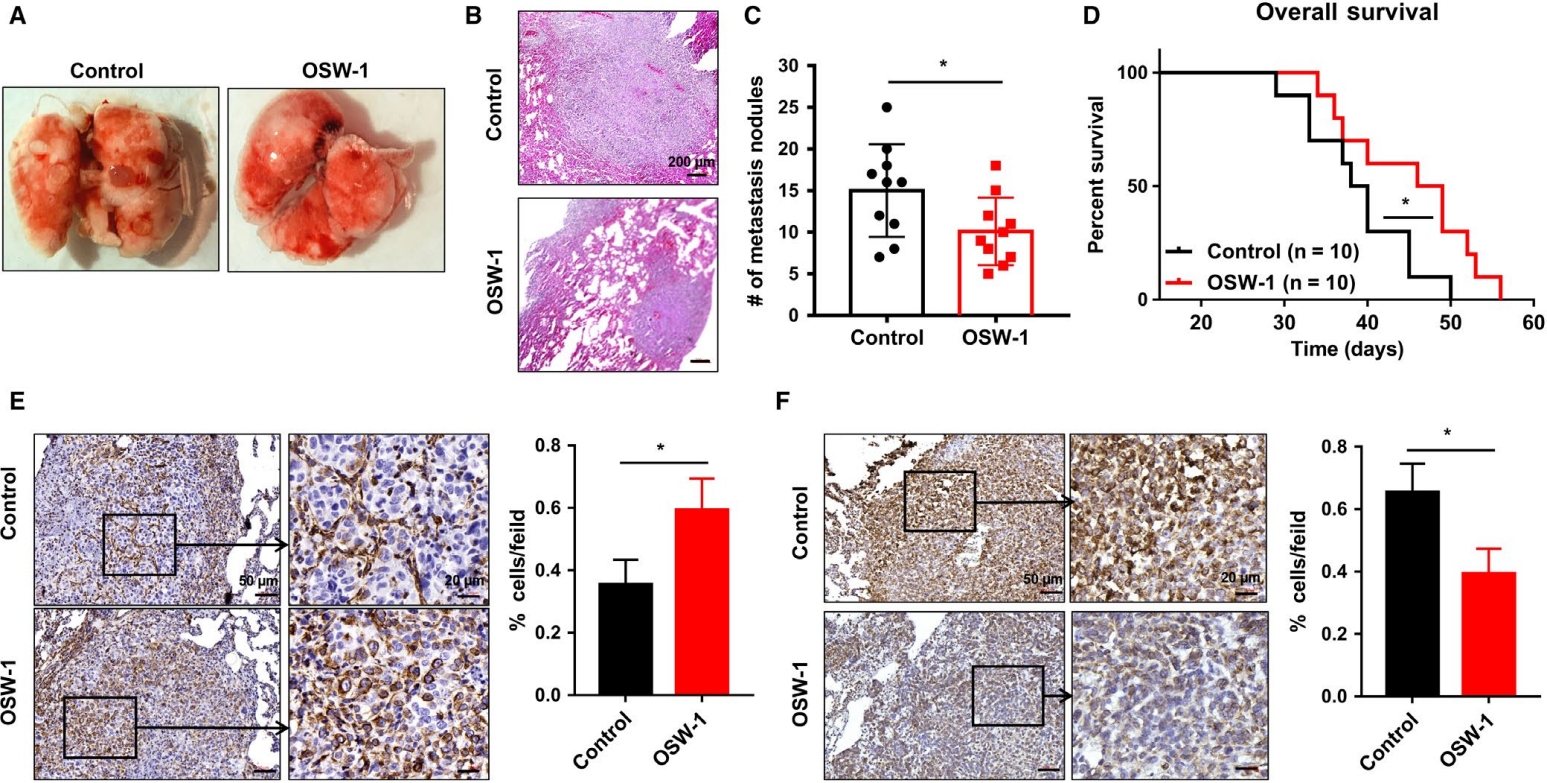
OSW‐1 reduces tumor metastasis in vivo. A‐C, Mice were treated with OSW‐1 and lungs were collected and counted. The lung metastatic nodules in OSW‐1 treatment group were obviously less than those in nontreated group. Hematoxylin and eosin staining further found that metastatic nodules in the OSW‐1 group were smaller than those in control group. Scale bar = 200 µm in (B). D, Survival analysis showed that the survival time of OSW‐1 treatment group (47.5 d) was longer than that of control group (39 d) (*P* < .05). E‐F, OSW‐1‐treated group showed remarkable upregulation of E‐cadherin and downregulation of Vimentin by immunohistochemistry assay. Scale bars = 50 µm in left (E) and (F), 20 µm in right (E) and (F). *P < .05

Finally, we sought to test whether OSW‐1 could inhibit TNBC metastasis in vivo. We tested OSW‐1 role in the metastatic setting in 4T1 (triple negative) mBC mouse model. To evaluate this, we implanted orthotopic 4T1 breast tumors in the mice and treated with OSW‐1 daily when the tumors reached 3 mm, then resected the primary tumor at a diameter of 1.0 cm, and initiated treatment 1 week after surgery. In our study, OSW‐1 resulted in significantly fewer metastatic nodules in lungs (Figure 5A‐C). In addition, the medium survival of control and OSW‐1 groups were 39, 47.5 days respectively and OSW‐1 treatment resulted in a longer survival compared with nontreated group (Figure 5D). Furthermore, OSW‐1 treatment showed a markedly increased E‐cadherin expression and decreased Vimentin expression as measured by immunohistochemistry assay (Figure 5E,F), suggesting that OSW‐1 inhibits metastasis mediated by EMT in vivo.

### 3.6 NFATc2 involved in OSW‐1‐induced cell death, migration and invasion and tumor suppression

**Figure 6:**
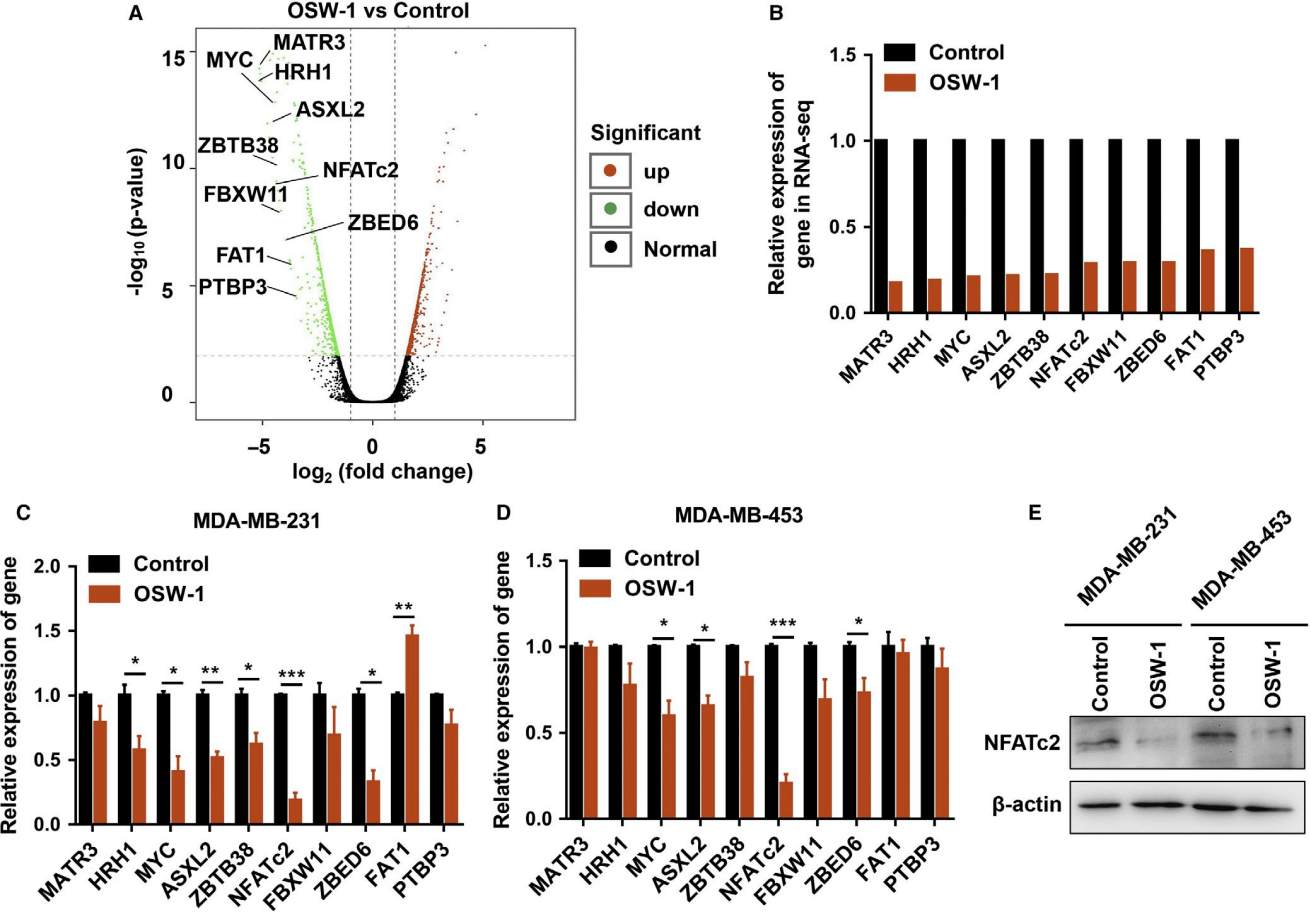
Genes in MDA‐MB‐231 cells are significantly changed by OSW‐1. A‐B, RNA sequencing was conducted to discover gene expression changes and some significant ones caused by OSW‐1 treatment. C‐D, We validated the expression of these genes in triple‐negative breast cancer (TNBC) cells exposed to OSW‐1 treatment (50 ng/mL for 24 h) by qRT‐PCR assay. E, NFATc2 was measured by western blotting in TNBC cells with OSW‐1 treatment (50 ng/mL for 24 h). *P < .05, **P < .01, ***P < .001

To interrogate the molecular mechanism of how OSW‐1 induced cell death, migration and invasion in TNBC, RNA‐sequencing was carried out to evaluate the altered gene expression in MDA‐MB‐231 treated with OSW‐1. We selected 10 genes associated with oncogenesis, and studied the top changed ones (*P* < .05, FC log_2_ > 2) with treatment of OSW‐1 (50 ng/mL for 24 hours) in MDA‐MB‐231 cells (Figure 6A,B). Subsequently, we validated the expression of these genes after OSW‐1 treatment (50 ng/mL for 24 hours) in MDA‐MB‐231 and MDA‐MB‐453 cells by qRT‐PCR analysis, and demonstrated that the expression of NFATc2 was the lowest one after treatment with OSW‐1 compared with non‐OSW‐1 group (Figure 6C,D). Furthermore, western blotting assay confirmed that NFATc2 was significantly lower in OSW‐1 group than control group (Figure 6E). So, we suspected that NFATc2 might be the key factor in OSW‐1‐induced cell death on breast cancer cells.

**Figure 7:**
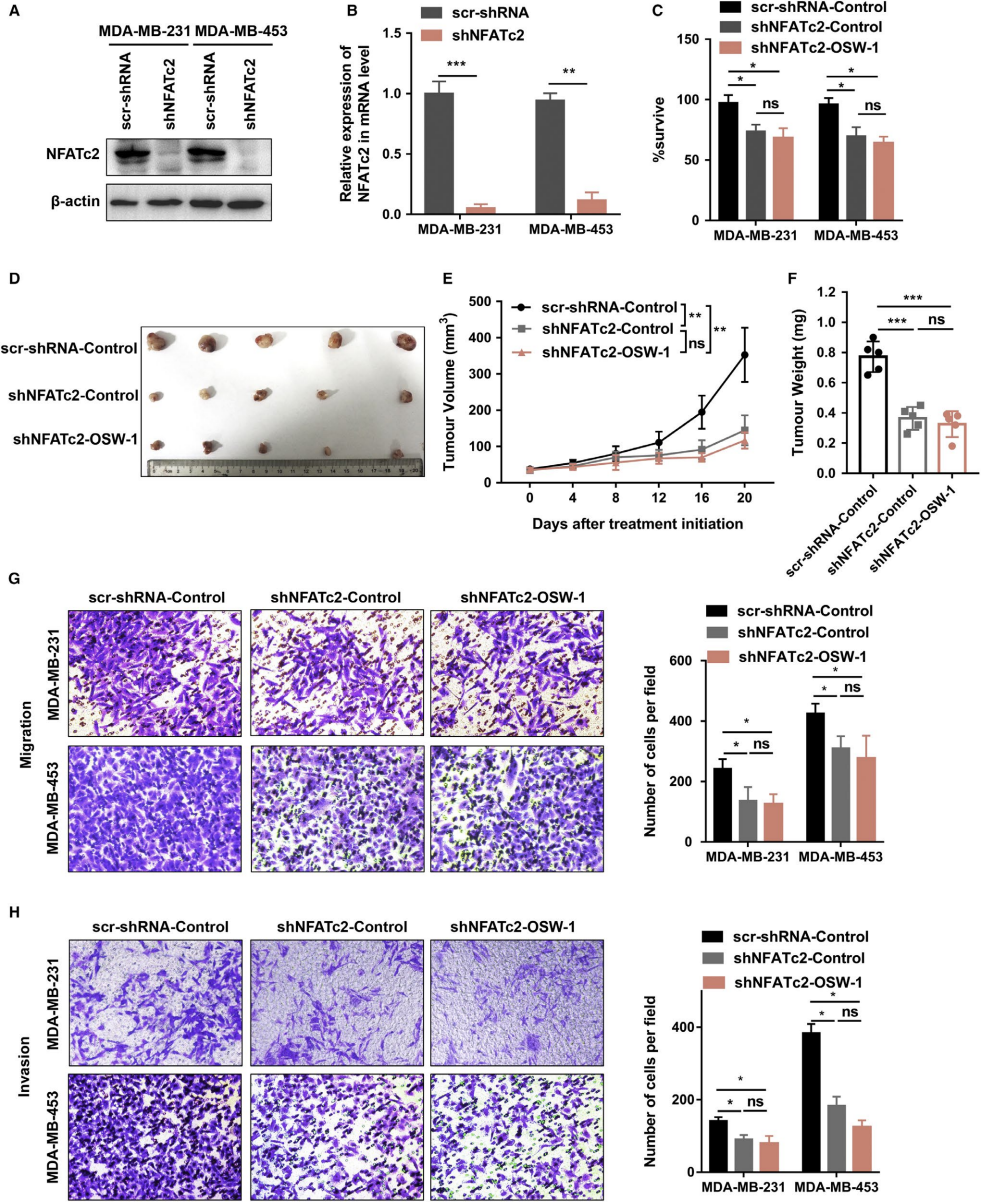
NFATc2 involved in OSW‐1‐mediated effects on apoptosis, migration and invasion, and tumor suppression. A‐B, Western blotting and qRT‐PCR confirmed knock downing of NFATc2 following transfection with NFATc2‐shRNA. C, MDA‐MB‐231 and MDA‐MB‐453 cells transfected with scr‐shRNA or shNFATc2 were treated with PBS or OSW‐1 (50 ng/mL) for 24 h and then examined by cell count kit 8 (CCK8) assay. D‐F, Removing subcutaneous tumors (MDA‐MB‐231 model) after OSW‐1 treatment for 20 d. G,H, The transwell assays showed that there was no difference in the number of migrated and invaded triple‐negative breast cancer cells between control and OSW‐1 group after knocking down of NFATc2. *P < .05, **P < .01, ***P < .001, ns, no significant

To ascertain the biological mechanism of NFATc2, NFATc2 targeted shRNA was used to block NFATc2 expression in both MDA‐MB‐231 and MDA‐MB‐453 cells. The levels of NFATc2 mRNA and protein expression were significantly reduced with shRNA‐cells, as compared to the blank cells (Figure 7A,B). Interestingly, knocking down of NFATc2 restrained cell proliferation compared with the control cells, while silencing NFATc2 significantly rescued TNBC cells from OSW‐1‐mediated cell death (Figure 7C). Similarly, OSW‐1 could inhibit tumor growth in vivo (Figure 3A‐C), on the contrary, suppression of NFATc2 also significantly blocked OSW‐1‐mediated tumor shrinkage (Figure 7D‐F). Moreover, knockdown NFATc2 expression decreased the migration and invasion of MDA‐MB‐231 and MDA‐MB‐453 cells in NFATc2‐depleted cells compared with the controlled cells (*P* < .05) (Figure 7G,H). However, there was no difference in the number of migrated and invaded TNBC cells between control and OSW‐1 groups after knocking down of NFATc2 (Figure 7G,H), suggesting that OSW‐1‐induced migration and invasion through NFATc2.

In summary, these results demonstrated that NFATc2 participated in OSW‐1‐induced cell death, migration and invasion and tumor suppression.

## 4 DISCUSSION

Recently, OSW‐1 has been reported to show strong antitumor activities in various tumors, including hepatocellular carcinoma, malignant brain tumors, ovarian cancer, pancreatic cancer, leukemia, and colon cancer.^10^, ^11^, ^12^ Interestingly, it shows less sensitive to nonmalignant cells, which is an important feature of antitumor drug.^13^ To elucidate the cytotoxic effect of OSW‐1 in breast cancer cells and normal endothelial cells, we examined cell viability using CCK8 assay and found that OSW‐1 has an extremely strong anticancer activity in breast cancer cells, but lower toxicity to normal cells.

Triple‐negative breast cancer is characterized as the deficiency of the expression of estrogen receptor, progesterone receptor, and human epidermal growth factor 2.^19^, ^20^ It looks more chemo‐sensitive than other subgroups of breast cancer, however, it is also characterized to have the most aggressive behavior and the poorest prognosis.^21^, ^22^, ^23^, ^24^ Once metastasized, TNBC has a high predisposition to important internal organs, including lungs, brain, and liver, ultimately resulting in a shorter OS than other subtypes.^25^, ^26^ Therefore, developing therapeutic treatments for TNBC is important for alleviating the burden of TNBC. In this study, we found that OSW‐1 restrains cell proliferation and metastasis both in vivo and vitro.

Recently, apoptosis, as a universal cellular process, has been confirmed to become a crucial mechanism of many anticancer drugs. Garcia et al have suggested that OSW‐1 could reduce mitochondrial membrane potential and the increase of cytosolic calcium and then induce calcium‐dependent apoptosis.^15^ Zhu et al showed that OSW‐1 induces apoptosis via caspase‐8‐dependent cleavage of Bcl‐2 in Chinese hamster ovary cells.^27^ Yan et al found that OSW‐1 inhibits colon cancer cells through the intrinsic apoptotic pathway.^12^ Therefore, we hold that apoptosis may be a vital element in OSW‐1‐induced cell death. To confirm our suppose, we performed Annexin V/PI‐labeled flow cytometric and TUNEL assays, and found that apoptosis significantly increases in OSW‐1‐treated TNBC cells.

Epithelial‐mesenchymal transition is defined as the transformation of the epithelial cells with a mesenchymal phenotype.^28^, ^29^, ^30^ In carcinomas, EMT is also known as epithelial cell plasticity and it usually begins with the loss of epithelial cell polarity and the breakdown of the E‐cadherin‐related cell‐cell adhesives.^28^ Acquisition of positivity for mesenchymal markers induces the increased activity of cancer cells and a high risk of lymph node or distant metastases. Here, we revealed that OSW‐1 treatment exhibits decreased migration and invasion ability, and that E‐cadherin expression increases and Vimentin expression decreases in OSW‐1‐treated groups both in vitro and in vivo, demonstrating that OSW‐1 could inhibit metastasis of TNBC mediated by EMT.

Recent researches have revealed that the calcium signaling transcription factor nuclear factor of activated T cells (NFAT) could influence cell proliferation, invasion, migration, angiogenesis, and stromal modulation in different tumors such as breast, colon, pancreas, and melanoma.^31^, ^32^, ^33^ Quang et al have described that NFAT is associated with TGFβ‐induced EMT.^34^ Therefore, NFAT contributes to malignant properties, including cell proliferation, invasion, migration, and EMT. Here, we revealed that knocking down of NFATc2 using shRNA significantly rescues TNBC cells from OSW‐1‐mediated effects on cell death, tumor growth, invasion and migration, indicating that NFATc2 is involved in OSW‐1 inhibition of TNBC progression.

In conclusion, this study showed that OSW‐1 has the inhibitory effects on the proliferation and metastasis of TNBC both in vitro and vivo. First, we showed that OSW‐1 has anticancer activity on breast cancer cells, but lower toxicity to normal cells. Accordingly, it also showed significant repression of tumor growth in xenograft model. Furthermore, we found that OSW‐1‐mediated cell death by activating apoptosis. To confirm if OSW‐1 also has the effect in TNBC cell migration and invasion, we performed transwell assays and revealed that the number of migrated and invaded TNBC cells is significantly decreased in OSW‐1‐treated group, which might be associated with EMT inhibition. In addition, OSW‐1 also could inhibit lung metastasis in 4T1 mouse TNBC model. Finally, to elucidate the molecular mechanism of OSW‐1 in TNBC, we performed RNA‐sequencing and cellular functions and identified NFATc2 as a pivotal factor for OSW‐1‐mediated effects on cell death, tumor growth, invasion, and migration. Our study suggests that OSW‐1 may be a new and promising strategy to treat TNBC.

## CONFLICT OF INTEREST

These authors have no conflict of interest to declare.

## AUTHOR CONTRIBUTIONS

Xiaorong Ding and Yongmei Yin designed the project. Xiaorong Ding, Yumei Li, and Jun Li performed the experiments and drafted the manuscript. Xiaorong Ding and Yumei Li analyzed the data. Yongmei Yin proofread and revised the manuscript. All authors read and approved the final manuscript.

## Data Availability

The data used to support the findings of this study are available from the corresponding author upon request.
